# Genetic Diversity and Demographic History of *Ganoderma boninense* in Oil Palm Plantations of Sarawak, Malaysia Inferred from ITS Regions

**DOI:** 10.3390/microorganisms7100464

**Published:** 2019-10-16

**Authors:** Frazer Midot, Sharon Yu Ling Lau, Wei Chee Wong, Hun Jiat Tung, Mui Lan Yap, Mei Lieng Lo, Mui Sie Jee, Simon Peter Dom, Lulie Melling

**Affiliations:** 1 Sarawak Tropical Peat Research Institute, Lot 6035, Kuching-Kota Samarahan Expressway, 94300 Kota Samarahan, Sarawak, Malaysia; sharolyl@sarawak.gov.my (S.Y.L.L.); yapmuilan@sarawak.gov.my (M.L.Y.); loml1@sarawak.gov.my (M.L.L.); jeemuisie@sarawak.gov.my (M.S.J.); simonpd@sarawak.gov.my (S.P.D.); luliem@sarawak.gov.my (L.M.); 2 Applied Agricultural Resources Sdn. Bhd. (AAR)—University of Nottingham Malaysia Campus (UNMC) Biotechnology Research Centre, Jalan Broga, 43500 Semenyih, Selangor Darul Ehsan, Malaysia; wongwc@aarsb.com.my (W.C.W.); tunghj@aarsb.com.my (H.J.T.)

**Keywords:** *Ganoderma boninense*, oil palm, basal stem rot, phylogenetic, phylogeographic relationship, ITS

## Abstract

*Ganoderma boninense* causes basal stem rot (BSR) and is responsible for substantial economic losses to Southeast Asia’s palm oil industry. Sarawak, a major producer in Malaysia, is also affected by this disease. Emergence of BSR in oil palm planted on peat throughout Sarawak is alarming as the soil type was previously regarded as non-conducive. Phylogenetic analysis indicated a single species, *G. boninense* as the cause of BSR in Sarawak. Information on evolutionary and demographic history for *G. boninense* in Sarawak inferred through informative genes is lacking. Hence, a haplotype study on single nucleotide polymorphisms in internal transcribed spacers (SNPs-ITS) of *G. boninense* was carried out. Sequence variations were analysed for population structure, phylogenetic and phylogeographic relationships. The internal transcribed spacers (ITS) region of 117 isolates from four populations in eight locations across Sarawak coastal areas revealed seven haplotypes. A major haplotype, designated GbHap1 (81.2%), was found throughout all sampling locations. Single nucleotide polymorphisms were observed mainly in the ITS1 region. The genetic structure was not detected, and genetic distance did not correlate with geographical distance. Haplotype network analysis suggested evidence of recent demographic expansion. Low genetic differences among populations also suggested that these isolates belong to a single *G. boninense* founder population adapting to oil palm as the host.

## 1. Introduction

Oil palm (*Elaeis guineensis* Jacq.) is a monoecious species of the Arecaceae family. Oil palm is the world’s leading source of vegetable oil and fat with palm oil and palm kernel oil accounting for approximately 81.85 million metric tons (40.2%) of the world’s vegetable oil supply in 2018 [1]. Malaysia and Indonesia are major palm oil producers in Southeast Asia and together produced 62 million metric tons (85%) of global palm oil production in 2018 [1]. In Malaysia, oil palm plantations, with a total planted area of 5.85 million hectares, produced 20.5 million metric tons of palm oil (25% of world palm oil supplies), with Sabah and Sarawak being the largest producers in the country [1,2]. As palm oil is the lowest priced oil [1], it is expected to continuously attract demand globally. Thus, the sustainability of the oil palm industry is critical to ensure continuous production. 

The sustainability of oil palm is threatened by pests and diseases. Among major pathogens, *Ganoderma boninense*, a fungus of the Basidiomycota division, is the most devastating pathogen which causes basal stem rot (BSR) and upper stem rot (USR) disease of oil palm [3,4]. It is estimated that by 2020, more than 60 million oil palm trees could be infected in Malaysia [5]. Upper stem rot (USR) exhibit similar field symptoms to BSR, with the only difference being the invasion site on the oil palm tree [4,6,7,8]. The USR is described as *G. boninense* infection at one metre from ground level and BSR as base level infection [8]. Ariffin et al. [9] identified *Ganoderma* species isolated from oil palm BSR as *G. boninense*. Subsequent studies reported that only three *Ganoderma* species, namely *G. boninense, G. miniatocinctum* and *G. zonatum*, associated with oil palm in Malaysia were determined to be pathogenic [10]. *Ganoderma applanatum, G. lucidum, G. pfeifferi*, *G. philippi* and *G. tornatum* were proven to be non-pathogenic to oil palm through artificial infection studies [10]. It is generally accepted that the *G. boninense* species remains a major disease threat and limiting factor for oil palm productivity [4,11,12,13].

Reports on BSR disease in various soil types are alarming and geographical distribution mapping of disease incidence such as in Selangor, Malaysia did not show any relationship to different types of soil series [14]. Although peat soil was once assumed to be non-conducive for BSR disease development [15], various studies regarding BSR and USR incidence in oil palm planted on peat have been reported [8,9,14,16]. Out of the total 5.85 million hectares of oil palm planted area in Malaysia, only 13% are planted on peat where most can be found in Sarawak [17]. However, regardless of soil type or any other environmental factors influencing disease development, failure to recognise the progression of BSR disease broad occurrence throughout this industry could translate into complications for next generation of planting. Disease incidence of BSR have been reported would continue to increase with subsequent replanting with younger palms exhibiting symptom [7,10]. One major hurdle in BSR disease management is that early detection can be difficult as affected oil palm can appear symptomless in early infection stage. The slow disease progression only adds to the complication as once foliar symptoms or basidiomata are detected, extensive infection could have been affecting the host for years [11,12,18]. *Ganoderma boninense* is a weak competitor for available substrate and requires a large inoculum size to be able to infect a susceptible host [18]. The mode of infection involves an initial biotrophic phase that is followed by aggressive necrotrophic phase when the pathogen begins to secrete extensive cell wall degrading enzymes to further invade and degrade plant tissues [18]. Formation of pseudosclerotia (melanised mycelium) within host tissues and the roots’ external was also suggested as a possible third phase [18]. 

Identification of the *Ganoderma* species has been difficult as they are genetically heterogeneous with a wide range of genetic variations caused by distinct geographical origins and outcrossing over generations [19,20]. Field conditions and the developmental stage of *Ganoderma* basidiomata can also influence the descriptions for species identification [21]. Regional variations in basidiomata morphological characteristics have been reported for *G. lucidum* isolates [22]. Falsely identified *Ganoderma* isolates can be resolved using molecular methods such as polymerase chain reaction (PCR) of internal transcribed spacers (ITS) region [23]. The multiplex PCR technique, which is less time-consuming compared to morphological assessment, has also been applied to identify *G. boninense, G. zonatum* and *G. miniatocinctum* [24,25]. However, this multiplex PCR protocol is not publicly accessible, and the form of the commercial kit is yet to be available from the Malaysian Palm Oil Board (MPOB).

Molecular approaches have been widely adapted in the field of biological science, including fungal identification in microbial ecology and plant pathology. Molecular methods were used to study and identify plant pathogens of many agricultural crops including oil palm [26]. With advancements in biotechnology, molecular genetic markers have been developed for rapid identification of fungi at multiple levels [26,27]. The development of gene-specific primers for PCR amplification [27] has facilitated detection and identification, as well as systematic studies of fungal pathogens. The nuclear ribosomal DNA (nrDNA) ITS, with its conserved feature within species and multi-copy number per genome, has universally been designated as the official barcode for fungi species level molecular identification [28]. The application of primers designed from the ITS1 region was useful for specific detection of phytopathogenic *Ganoderma* isolates [29].

Likewise, alternative approaches to the taxonomic problems in *Ganoderma* have arisen through nrDNA sequences analysis [7,21]. Morphological characteristics of *Ganoderma* could differ due to environmental factors so they are unreliable as the single criterion to distinguish diverse species. As such, the informative aspect of molecular characteristics should be investigated [30]. As disease management is important for sustainability of the oil palm industry, differentiation and understanding of individuals, or subpopulation epidemiology, would be useful to elucidate spatial disease establishment mechanism and spread. Hence, this study sets out to (i) compare the level of polymorphism of ITS sequences, (ii) determine the haplotype diversity and distribution and (iii) infer the phylogenetic relationship of sampled *Ganoderma* isolates. It is essential to understand the current population structure of pathogenic *Ganoderma* spp. to predict the future population evolution and to resolve controversies regarding its taxonomic position. 

## 2. Results

### 2.1. Sequence Variations

A total of 117 *G. boninense* ITS sequence data sets were obtained, representing four *G. boninense* populations in oil palm plantations. The combination of ITS1 and ITS4 primer amplified regions from the 3′ end of 18S rDNA to the 5′ end of the 28S rDNA included the ITS1, 5.8S rDNA and ITS2 regions. After sequence alignment and trimming, ITS1F primer read sequences were trimmed based on ITS1 primer reads. The final sequence length was 601 bp with six variable sites, five parsimoniously informative sites and 595 bp conserved sites were identified. The mean total nucleotide composition was 30% thymine (T), 23.7% cytosine (C), 22.1% adenine (A) and 24.1% guanine (G). Annotations for *G. boninense* were done based on information available in Bridge et al. [31]. Multiple alignments of the available record of *G. boninense* sequences in GenBank^®^ which consisted of a partial 18S small subunit gene and complete ITS1 region sequence revealed that our samples were missing 13 nucleotides from the 3′ terminus of the 18S small subunit gene. The length of our partial ITS1 region was 198 bp, the 5.8S was 155 bp and the complete ITS2 region was at 198 bp within the trimmed sequence. Sequences from ITS1F reads contained the complete ITS1-5.8S-ITS2 region. No sequencing errors or random substitutions were detected in the studied region. The haplotype search was carried out from 1st nucleotide position to 601. Seven haplotypes with one unique sequence and another six sequences shared among populations were distinguished based on single nucleotide polymorphisms of ITS region (SNPs-ITS) (Table 1). Overall, six substitutions were found among the haplotypes which include one transversions and five transitions. The 5.8S rDNA region was conserved throughout all samples. Based on obtained sequences, partial sequence of ITS1 was more diverse than ITS2 with the ITS1 region contained four transitions and one transversion while ITS2 region only has one nucleotide substitution in the form of transition (Table 1). However, as only partial sequences of ITS1 region were obtained, it is also possible that the 13 missing nucleotides could consist of additional substitution.

Ninety-five isolates with GbHap1 corresponding to 81.2% of total individuals were found in all populations. Samples from Matu-Daro contained the highest number of individuals with GbHap1. Details on the haplotype distribution in each population are shown in Table 2. The Matu-Daro population also contained the highest number of haplotypes with six haplotypes detected. Samples from Asajaya were only represented by a single haplotype. Balingian and Miri populations contained five and four haplotypes, respectively (Table 2).

### 2.2. Phylogenetic Relationships among Haplotypes

**Figure 1:**
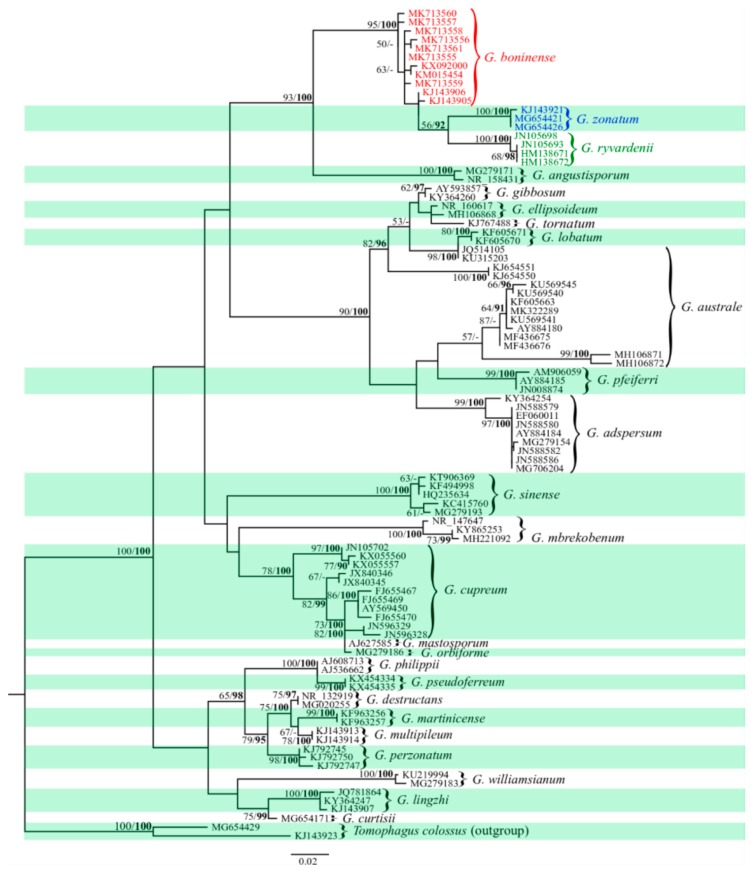
Phylogenetic tree constructed for *G. boninense* (species highlighted in red font) based on maximum likelihood (ML) and Bayesian phylogenetic analysis of internal transcribed spacers (ITS) sequences. Statistical values shown are ML-bootstrap values above 50% and Bayesian inference (BI) posterior probability above 90% (in bold). The scale bar (0.02) indicates the number of nucleotide substitution per site. Sub-clusters of *G. ryvardenii* and G. *zonatum* are highlighted in green and blue font, respectively. *Tomophagus colossus* is included as the outgroup. Data matrix and tree files can be viewed through the following URL: http://purl.org/phylo/treebase/phylows/study/TB2:S24430

Phylogenetic analyses through ML and BI revealed similar tree topologies. *Tomophagus colossus* (KJ143923 and MG654429), which is closely related to *Ganoderma* genus in the Ganodermataceae family was included as an outgroup in the phylogenetic analysis [32]. The sequences obtained from NCBI GenBank^®^ that were used in phylogenetic analysis is shown in Table S1. A summary of the phylogenetic relationships among haplotypes of *G. boninense* is illustrated by the ML tree (Figure 1). Monophyletic status of *G. boninense* from other *Ganoderma* species was strongly supported by both ML and BI. Samples of *G. boninense* from Sarawak formed a cluster with *G. boninense* originated from Peninsular Malaysia (KM015454 and KX092000) and Japan (KJ143905 and KJ143906) within the *G. boninense* clade (Figure 1). The sub-cluster of *G. zonatum* and *G. ryvardenii* within the *G. boninense* clade formed sister clades.

### 2.3. Population Structure Analyses

Each distinct haplotype was treated as an allele for population structure analyses. Nucleotide diversity among the seven *G. boninense* populations was low, ranging from 0.001 (Matu-Daro and Balingian) to 0.002 (Miri). On the other hand, the haplotype diversity ranged from 0.343 (Matu-Daro) to 0.561 (Miri) (Table 2). The nucleotide diversity and haplotype diversity of the Asajaya population were represented by only a single haplotype. Pairwise genetic distances among the seven haplotypes of *G. boninense* and one outgroup (*Tomophagus colossus*) are showed in Table S2. To sum up, genetic distance values were low among the seven haplotypes of *G. boninense*, ranging from 0.2% to 0.7%. In contrast, higher genetic distance values (11.7–12.0%) were obtained with the presence of an outgroup. The pairwise F_ST_ values and results of the Chi-square tests for genetic differentiation among populations are displayed in Table 3. Low F_ST_ values were obtained for Asajaya compared to all populations with *p* > 0.05. Negative F_ST_ values for Miri, Matu-Daro and Balingian with *p* > 0.05 indicated that these populations shared similar genetic material. High gene flow (migration index) with Nm > 4 suggested that these populations behave as a single panmictic unit. The AMOVA result revealed a variance of 100.254% within *G. boninense* populations whereas a negative percentage of variations (−0.254%) was presented among *G. boninense* populations (Table 4). As no genetic structure was detected in AMOVA, a Mantel test was conducted to assess any possibility for isolation-by-distance phenomenon with up to a 504 km distance between isolates. The Mantel test, however, revealed no significant differences between genetic and geographic distances with an Rxy value of 0.010 and a *p*-value of 0.411. Similar to the Mantel test, no spatial structure was detected through the spatial autocorrelation test with *p* > 0.01 (*p* = 0.021) within the sampled populations.

The *Ganoderma boninense* population within Sarawak based on F_ST_ values, AMOVA, the Mantel test and spatial autocorrelation analysis of SNPs-ITS did not detect any genetic structure and no clear differences were found among populations within the sampling locations.

### 2.4. Demographic History of G. boninense Populations

Tajima’s D test compares allelic frequency of segregating nucleotide sites and a significant positive value indicates low levels of both low and high frequency polymorphism while a significant negative value indicates an excess of low frequency polymorphisms which suggest recent population expansion [33]. Fu’s FS test is based on haplotype frequencies; a significant positive value would translate as deficiency of alleles to indicate a recent population bottleneck while a negative value would describe an excess number of alleles which is a signature of recent population expansion or genetic hitchhiking [34]. The neutrality test determined Tajima’s D and Fu’s F_S_ values as negative with non-significant *p*-values (Table 2). A negative Tajima’s D and Fu’s F_S_ indicates a recent population expansion with excess number of alleles. Despite that, the null hypothesis of neutrality for our *G. boninense* ITS marker was not rejected as Tajima’s D and Fu’s F_S_ values obtained were not statistically significant. Thus, demographic expansion history based on the neutrality test could not be concluded.

**Figure 2:**
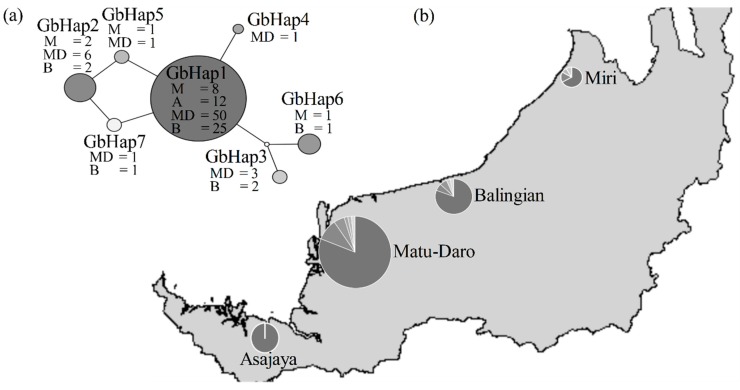
(**a**) Haplotype network of *G. boninense* based on ITS sequences. Radius of the circle for the haplotype network is proportional to the number of isolates obtained, as shown in the parentheses. The major haplotypes (GbHap1 and GbHap2) are placed in the inner clades. The small circle is a hypothesised missing haplotype. (**b**) Geographical distribution of *G. boninense* haplotypes according to designated population: Miri (M), Asajaya (A), Matu-Daro (MD) and Balingian (B). The number of specific haplotypes found in a certain location is shown in the haplotype network.

As supplementary, haplotype network was generated with TCS 1.21 (Figure 2). The generated haplotype network displayed a star-like structure with a missing haplotype linking GbHap3 and GbHap6 to the central GbHap1. The major haplotype (GbHap1) that can be found in multiple locations formed interior clade among the haplotypes and could be more ancestral than other haplotypes. Minority haplotypes positioned one step away could have been derived from the major haplotype. Based on coalescent theory, these haplotypes could be the product of recent mutation events.

## 3. Discussion

### 3.1. High Morphological Plasticity in Ganoderma spp. Leads to Misidentification

All samples collected in this study were identified as *G. boninense* isolates based on ITS DNA sequences. Although Rakib et al. [8] identified randomly sampled pathogenic *Ganoderma* isolates infecting oil palm using multiplex PCR as *G. zonatum*, *G. boninense* and *G. miniatocinctum*, it is however, generally accepted that *G. boninense* is the dominant causal phytopathogen of BSR and USR disease in Malaysia [11,12]. Nucleotide database of NCBI showed that the only ITS record of *G. miniatocinctum* (Accession No. KM220586) from Malaysia is identical to *G. boninense* (Accession No. MG200173). The record of *G. zonatum* ITS from Malaysia, however, was not publicly available for comparison with the generated *G. boninense* clade haplotypes. Phylogenetic analyses verified the inclusion of generated haplotype sequences within the *G. boninense* clade. Phylogenetic studies have also demonstrated that *G. boninense* and *G. zonatum* commonly formed a unique clade and are closely related to one another than other *Ganoderma* species [21,35]. 

Phylogenetic analysis revealed a strong support for clade containing *G. angustiporum*, *G. boninense*, *G. ryvardenii* and *G. zonatum* (Figure 1). However, only species such as *G. boninense*, *G. ryvardenii* and *G. zonatum* indicated host preference to palm (Table S1). Our results are in agreement with Pilotti’s report [4] which demonstrated that *G. boninense* has a restricted host range to palm in natural condition. In the literature, non-palm hosts for *G. boninense* have not been reported. Apart from oil palm, other palm host species for *G. boninense* also include *Clinostigma savoryana* (pacific beauty palm), *Cocos nucifera* (coconut), *Livistona chinensis* (fountain palm) (var. *boninensis* and var. *subglobosa*) and *Wodyetia bifurcate* (foxtail palm) [36]. The first record of disease occurrence for foxtail palm in Malaysia was reported in 2018, indicating that this pathogen can be a threat to both oil palm industry and ornamental palms used in landscaping [37]. Elliott et al. [38] reported 12 different palm species (native and non-native) that could host *G. zonatum* and the non-palm host records may have been inaccurately identified based on information from phylogenetic analyses. *Ganoderma ryvardenii* is a relatively new species compared to *G. boninense* and *G. zonatum* and has only been reported in oil palm plantation from Cameroon [39]. *Ganoderma angustisporum* is a new species reported in 2018 by Xing et al. [40] in China that differs in morphological features and host tree compared with *G. boninense, G. zonatum and G. ryvardenii*. It is plausible that the genus *Ganoderma* is relatively young and expanding its range to other hosts or climate zones [41] and many species have yet to be discovered. These unknown species could assist in explaining *Ganoderma* species divergence.

High morphological plasticity is common among *Ganoderma* species and this can lead to species misidentification [7,23]. A survey of 600 *Ganoderma* spp. sequences in the NCBI GenBank^®^ nucleotide database has revealed that 65% of these sequences were misidentified or incorrectly labelled [41]. Hence, reliable taxonomic assignments in public databases would benefit researchers across multiple disciplines and, given the economic impacts of *Ganoderma* species such as *G. boninense* in the oil palm industry, accurate identification would influence disease management. Geographical regions and fungal-host relationships should also be considered in assessing monophyletic taxa in the *Ganoderma* genus [42].

### 3.2. Ganoderma boninense SNPs-ITS Variation Analyses Showed a Single Population Throughout Sarawak

Application of ITS in haplotypes studies have been applied to fungal pathogen such as *Magnaporthe oryzae* [43] and *Fusarium incarnatum* [44]. Variations among *M. oryzae* was indicative of genome plasticity for adaptation to different agro-climatic zones [36]. The DNA polymorphism indices of ITS sequences for *F. incarnatum*, however, were less informative and the genetic variation was better resolved through haplotype studies of translation elongation factor gene (*tef1-*α) sequences [44]. Nuclear rDNA ITS region is considered as a fast evolving region [27]. Intra-strain heterogeneity in the ITS sequences has been reported for species such as *G. applanatum* var. *gibbosum*, *G. fornicatum*, *G. japonicum*, *G. lucidum* and *G. neojaponicum* [45,46]. Sequence variation within strains varies and may occur within ITS1, ITS2 or the 5.8S region.

In this study, *G. boninense* was further elucidated with analyses of SNPs-ITS (Table 1) and low genetic distances were found among the haplotypes (0.2–0.7%, Table S2). Furthermore, high genetic similarity and low genetic heterogeneity was found among all of the *G. boninense* populations analysed. This was reflected by low nucleotide diversity and low haplotype diversity with low number of polymorphic loci (0–5) (Table 2). The possibility of these populations belonging to a single panmictic unit can be drawn from the absence of genetic structure through negative F_ST_ values with high *p*-values (*p* > 0.05), high gene flows (Nm > 4) and genetic variations that were attributed to within the population (AMOVA). Current findings could indicate that these populations are from surviving individuals of *G. boninense* that were able to infect and adapt to oil palm. 

Identical ITS haplotypes of *G. zonatum* [38] and *G. applanatum-australe* [47] have been found across long distances and could best be explained by airborne basidiospores dispersal. Although there was evidence regarding the sub-population of *G. applanatum-australe* complex between Southern and Northern Hemisphere samples [47] with a wide host range, *G. zonatum* isolates throughout Florida, USA [38] revealed the absence of species complex or sub-population coupled with limited host family preference to Arecaceae (palm) without specificity to any host genus or species. Analysis on SNPs-ITS variation of *G. boninense* in this study did not detect the genetic structure as observed variation did not reveal any sub-population. This would suggest that only a single population of *G. boninense* can be found throughout the state of Sarawak. Population genetic structure analysis with ITS regions should, however, be interpreted with caution because ITS regions are multi-copy within the genome. Any variation which arises could potentially be credited to variation among repeats. Heterozygous sites within the ITS sequences could also potentially be chromosomal variation of paralogs and might not represent nucleotide variations from different chromosomes [48]. However, intra-strain and intra-species ITS sequence variation is not uncommon within the *Ganoderma* genus and has been used to delineate isolates within a species into geographically distinct groups [45,46]. The genetic structure of *G. boninense* in Sarawak could be re-examined to determine precisely the inclusion of more polymorphic gene markers such as β-tubulin or *tef1*-α for inferring phylogeography and the population structure of fungi [49].

Although high gene flow (Nm > 4) was calculated between populations in Sarawak, caution should be practiced in interpretation as any indication of an outcrossing preference by *G. boninense* as a calculation was made based on a single gene. Outcrossing is a common feature in basidiomycete sexual reproduction and the *G. boninense* gene flow inferred through microsatellites analysis which can extend to a regional level [50]. Fungi in high-density planting over large spatial scales with reduced host genetic heterogeneity are highly conducive for rapid adaptive evolution and distribution of fungal plant pathogens [51]. Hence, the monoculture nature of the oil palm plantation is an ideal platform for *G. boninense* to evolve and establish itself. Oil palm monoculture expansion could influence indigenous *G. boninense* population size and accelerate natural selection for the oil palm specific pathogenic strain. In North America, *G. zonatum* were exposed to native and non-native palms cultivations. They were found to evolve accordingly through generations of outcrossing and increases in genetic diversity but they are maintained as a single population [37]. However, research is still needed to validate the *G. zonatum* species existence outside of the Americas continent to ascertain global demographic expansion. Founder populations of any fungi would possess low genetic diversity with traces of genetic drift, but the combination of sexual reproduction and an increase in host availability could likely increase pathogen evolutionary potential through recent population expansion, as reported for *Alternaria brassicicola* [52]. In addition, samples in our study did not indicate any sub-population through the Mantel test and spatial autocorrelation with the farthest distance between isolates being 504 km. Similar alleles for *G. boninense* can also be found up to 700 km across regional geographical boundaries as reported in Merciere et al. [50] which support broad scale basidiospores dispersal.

### 3.3. Haplotype GbHap1 as a Dominant Ancestral Isolate in Recent Population Expansion

The demographic history of the *G. boninense* population in Sarawak was investigated using a neutrality test through Tajima’s D and Fu’s F_S_. Values were calculated as an individual population as well as a single population with both having negative values but were not statistically significant to conclude a demographic expansion (Table 2). The neutrality test based on haplotype frequencies such as Fu’s FS performed better than Tajima’s D in detecting demographic expansion, contraction and bottlenecks [53]. Fu’s FS test, however, can be affected by recombination events [53]. We did not perform additional tests for detection of recombination events as both neutrality tests were not significant. Simulation of demographic expansion for *G. boninense* samples from Peninsular Malaysia and Sumatra Island of Indonesia by Merciere et al. [50] through microsatellites suggested a demographic expansion time estimated to have occurred between 12,800 to 22,820 years ago. Authors, however, caution that population size and generation time could still be influenced by the introduction of new planting materials and the presence of actively sporulating *G. boninense* basidiomata. Regardless, population distribution and the expansion of sampled *G. boninense* isolates between Peninsular Malaysia and Sumatra, Indonesia was not influenced by factors such as planting generation, planting material genetic background or even regional geographical separation by way of the strait of Malacca [50]. Specific interaction between *G. boninense* isolates to its host (oil palm) was not detected through their microsatellites analyses and molecular evidence was insufficient to establish the existence of any sub-population. As multiple individuals have the potential to cause further infection and propagate, population size would continue to expand through outcrossing and basidiospores spreading across long distances by nature or human activities [54].

Although our study found no statistically significant evidence for demographic expansion using the neutrality test, a haplotype network was generated (Figure 2) and it displayed a star-like structure with a numerically dominant haplotype in the centre and multiple variants of haplotypes with low nucleotide diversity surrounding the central haplotype. Traces of species history that could alter its genetic makeup can be detected by studying haplotype and nucleotide diversity, haplotype frequencies and their pattern of distribution in order to explain possible demographic events that shaped the variations found within any population genetics through coalescent theory [47]. The relationship between haplotypes can also be better visualised through a network as reticulation events with ancestral and descendant co-existence are shown [55]. Reticulation events can occur in genetic clines at contact zones in the form of SNPs and in lineage-scale migration observed through allopatric populations [56]. This star-like configuration of the haplotype network could be indicative of a recent population expansion throughout the taxa’s history. However, estimation of expansion time was not possible through haplotype network visualisation. As coalescent theory suggests, haplotype numbers could only be an indicative that *G. boninense* isolates are recent individuals adapted to oil palm as hosts with GbHap1 being the numeric dominant haplotype as the ancestral isolate. Nevertheless, the haplotype network (Figure 2) configuration also included the possible missing haplotype and reticulation within the network—this could as well suggest that the sampled population may not represent actual haplotype diversity. Therefore, haplotype diversity of *G. boninense* may be higher than the information presented (Table 2). 

### 3.4. Challenges and Recommendations

The limited number of samples collected from each population might have resulted in underestimation of the actual haplotype distribution of *G. boninense* in Sarawak as samplings were conducted by collecting available basidiomata during sampling time without periodic resampling at the same location. 

Pilotti et al. [20] previously proposed a disease cycle time of four to seven years for a new generation of *G. boninense* to manifest based on mating type alleles ratio surveyed throughout 1998 to 2000 in Papua New Guinea. Their results also support the importance of basidiospores in long-distance dispersal. Isolates that share the same alleles could have derived from indigenous ancestors that have adapted to oil palm as their host through natural selection process. Sampling over time and the surrounding area of occurrence should provide insight into determining whether *G. boninense* is a palm-specific pathogen and disease severity correlation with subsequent replanting on diseased area. 

High genetic heterogeneity is common within the *G. boninense* population that has gone through generations of outcrossing [13,20,30,54]. Our observation, however, only revealed low genetic diversity of isolates that belong to a single population for Sarawak. Oil palm planting on peat in Sarawak started in the 1990s when available mineral soil for commercial agriculture development became limited [57]. Our sampling sites are first generation planting and the disease occurrence was random. All plantations were previously secondary forest and possibly contained low disease pressure. The random infection and low genetic diversity could suggest these isolates as founding population adapting to oil palm as host. Therefore, further spatio-temporal population structuring and genetic variation analyses on other informative gene markers are needed to determine pathogen evolutionary status. Plant pathogen genetic structure can affect host breeding programmed towards developing efficient genetic resistant material for disease control management [58]. 

In this study, the genetic structure based on a single-gene region was not found among the *G. boninense* obtained from study areas spanning a distance of approximately 504 km. Although the result may be because of the reliance on the analysis of a single gene region, this finding is nonetheless similar to the result reported by Merciere et al. [50], in which there was no genetic structure detected in the 357 isolates collected from Peninsular Malaysia and Sumatra, Indonesia genotyped using 11 genomic SSR markers. Construction and interpretation of any species demographic history should also be exercised with caution as application of different gene markers can lead to different population taxonomic histories [59]. This is due to the fact that demographic population expansion can also be associated with climatic events linking lineage diversity, expansion range and speciation. Variables such as sequence polymorphism level, sampling scheme, sample size, natural selection and mutation rate estimation can contribute to error in demographic history reconstruction [60]. The inclusion of several gene sequences can nonetheless reduce potential discrepancies due to randomness among loci [61]. Multiple loci gene analysis on genomic regions such as ITS regions, *tef1-*α and RNA polymerase II subunit 2 (*rpb*2) have been used within the *Ganoderma* genus, in elucidating phylogenetic analysis of closely related *Ganoderma* isolates to determine correct speciation and possible species complex formation [38,41]. Observation for *G. zonatum* in Florida, USA, however, has only shown that *rpb2* as the only gene region with significant differences while ITS region that was expected to contain higher genetic variability did not. Therefore, the multi-locus gene sequence for population genetic structure analysis may be relevant but not necessarily conclusive in describing the *G. boninense* population.

## 4. Materials and Methods 

### 4.1. Sampling Location and Sample Collection

**Figure 3:**
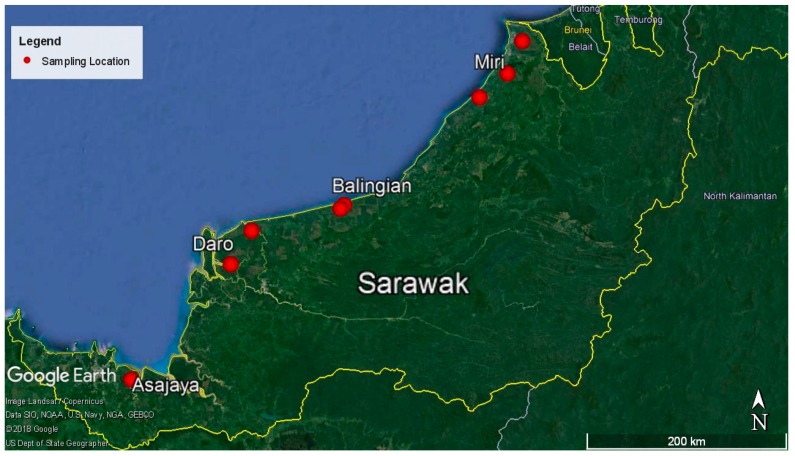
Sampling locations of *Ganoderma* basidiomata across oil palm plantations in Sarawak. Map extracted from Google Earth [62].

A total of 117 *G. boninense* basidiomata were collected from 107 BSR and 10 USR standing infected oil palm trees planted on peat in eight locations throughout Sarawak. These locations were then grouped together as local population representing their district or sub-district for analysis (Figure 3 and Table S3). A single basidiocarp was randomly collected from each diseased oil palm tree and treated as a single isolate. Field samples were brought to the laboratory for pure culture isolation. The surface of the basidiomata was cleaned with 70% (v/v) ethanol prior to isolation.

### 4.2. Ganoderma Culture

*Ganoderma* cultures were isolated from the basidiomata context using *Ganoderma* selective medium (GSM) as described by Ariffin and Idris [63]. Pure cultures (dikaryotic) were maintained on malt extract agar (MEA) prior to genomic DNA extraction. *Ganoderma* mycelia agar plugs were stored in sterile water as preservation by submersion in water would maintain *G. boninense* pathogenicity [64].

### 4.3. DNA Extraction, PCR Amplification and Sequencing

Cultures were grown for seven days in malt extract broth (MEB) before being harvested. The mycelia were snap-frozen in liquid nitrogen and ground to fine powder before DNA extraction [25]. Total genomic DNA was extracted from each sample using a modified cetyl-trimethylammonium bromide (CTAB) method in the presence of proteinase K [65]. 

The ITS region was amplified via PCR with the universal primer pair of ITS1 (5′-TCCGTAGGTGAACCTGCGG-3′) and ITS4 (5′-TCCTCCGCTTATTGATATGC-3′) adapted from White et al. [27] as well as ITS1F (5′-CTTGGTCATTTAGAGGAAGTAA-3′ and ITS4B (5′-CAGGAGACTTGTACACGGTCCAG-3′) adapted from Gardes and Bruns [66]. Components for PCR reaction were from Promega (Madison, WI, USA) unless stated otherwise. Template DNA (1.0 μL) was amplified in a reaction mixture containing 6.0 μL of 5× Colourless GoTaq^®^ Flexi buffer, 2.4 μL of MgCl_2_ (25 mM), 2.4 μL of dNTPs (2.5 mM), 0.75 μL of each primer (20 µM), bovine serum albumin (BSA) (10 mgmL^–1^) (Sigma-Aldrich, St. Louis, MO, USA) and 0.15 μL of Go*Taq*^®^ DNA polymerase. The reaction mixture was then adjusted to a final volume of 30 μL with nuclease free water. The thermal cycling parameters set on the C1000 Touch^TM^ Thermal Cycler (Bio-Rad, Hercules, CA, USA) were as follows: Initial denaturation at 95 °C for 2 min; 30 cycles of denaturation at 95 °C for 60 s; annealing at 50 °C (ITS1-ITS4 pair) for 30 s or 55 °C (ITS1F-ITS4B pair) for 50 s; extension at 72 °C for 60 s; and a final extension at 72 °C for 5 min. The PCR products were subjected to electrophoresis on 2% (w/v) agarose gel, performed at 75 V for 30 min, stained with ethidium bromide and visualised under UV light through a Molecular Imager^®^ Gel Doc™ XR^+^ Imaging System (Bio-Rad, Hercules, CA, USA). The PCR products were purified using a QIAquick PCR Purification kit (Qiagen, Germantown, MD, USA). A total of 107 samples were amplified with ITS1F-ITS4B and only 10 samples (six from Kuala Igan and four from Matu-Daro) were amplified using the ITS1–ITS4 primer pair. All purified PCR products were sent to First BASE Laboratories (Apical Scientific, Seri Kembangan, Selangor, Malaysia) for Sanger sequencing using the BigDye^®^ Terminator v3.1 Cycle Sequencing Kit (Applied Biosystems, Life Technologies Corporation, Austin, TX, USA) with forward primer. Single directional sequencing was used as Sanger sequencing method is able to amplify the entire ITS1-5.8S-ITS2 region with ITS1F.

### 4.4. Data Analyses

Chromas 2.4.4 (Technelysium) software was used to view the sequences and chromatograms. Multiple alignments of the sequences were conducted using the ClustalX 2.0.10 [67] and the alignments thereafter were inspected manually. Haplotype were generated by exporting the aligned sequences to FaBox [68]. *Ganoderma boninense* haplotypes were numbered in the order of their appearance in the analysis. Pairwise genetic distance between haplotypes was calculated in MEGA 7.0 with the Kimura 2-parameter evolution model (default setting) [69]. 

The levels of ITS variation within sampled *G. boninense* populations were examined by computing nucleotide diversity with Jukes–Cantor correction (PiJC) and the haplotype diversity indices (Hd) with DnaSP 6 [70]. The level of population subdivision (FST) between populations and the Chi-square probability test for population differentiation of the datasets were estimated using DnaSP 6 with 1000 permutations [70]. Neutrality tests such as Tajima’s D and Fu’s FS were also performed using DnaSP 6. Various neutrality tests such Tajima’s D [33] and Fu’s FS [34] were developed to detect DNA sequence variability that either accept or reject null hypothesis of neutral theory of evolution [71]. Both tests are commonly used to investigate the effects of demographic changes. The significance of spatial variation in the genetic diversity of *G. boninense* populations was calculated via hierarchical analysis of molecular variance (AMOVA) implemented in Arlequin 3.5 [72]. A Mantel test was used to further test for effect of isolation by distance based on correlation between genetic and geographical distances using 9999 permutations. A spatial autocorrelation test on correlation between genetic similarity and spatial distance classes was also tested with 9,999 permutations and 10,000 bootstraps. Both the Mantel test the and spatial autocorrelation were implemented in GenAlEx 6.5 [73].

Sequences representing each haplotype were deposited in NCBI GenBank^®^ corresponding to the accession number MK713555 to MK713561. Phylogenetic relationships were inferred by constructing a maximum likelihood (ML) tree using PhyML 3.1 [74] and Bayesian inference (BI) with MrBayes 3.2.7 [75], applying the Hasegawa–Kishino–Yano (HKY) nucleotide substitution model with gamma-distributed rate variation across sites and proportion of invariable sites selected through jModelTest 2.1.10 [76]. Phylogenetic confidence for ML was estimated by bootstrapping with 1000 replicate datasets and 2,000,000 generations for BI posterior probability. Phylogenetic trees were visualised in FigTree 1.4.4 [77]. Additionally, a haplotype network was constructed for visualisation of the relationship between haplotypes using TCS 1.21 [78] and tcsBU [79]. Annotations on the phylogenetic tree and haplotype network were edited using Inkscape 0.92.4 [80].

## 5. Conclusions

This study shed light on the phylogenetic and phylogeographic relationships of *G. boninense* isolates collected throughout oil palm plantations planted on peat located near coastal areas in Sarawak. *Ganoderma boninense* was identified as the causal agent regardless of BSR or USR origin. Analysis of variation in SNPs-ITS region showed a common major *G. boninense* haplotype distributed throughout Sarawak. Minority haplotypes were generated from this major haplotype based on haplotype network. Genetic structure was not detected among population with distribution of similar haplotype can be found up to the farthest distance of 504 km between sampling points. Haplotype diversity and the level of variations observed in the ITS region would suggest the possibility of only a single founder population of *G. boninense* existing in the collected samples throughout Sarawak. These findings, however, may only be related to the analysed gene of collected samples and should not be the representative patterns of the whole genome or taxa. Taking into consideration the disease cycle, subsequent resampling at the same or nearby location could also provide more information on population generation, population expansion and genetic differentiation of indigenous *G. boninense* isolates. Future application of comparing multiple gene regions such as *rpb*2 and *tef*1α could also be useful in resolving evolutionary relationships and population dynamics among phytopathogenic *G. boninense* or closely related species.

## Figures and Tables

**Table 1:** Variable sites of seven haplotypes of *G. boninense*.

	Variables	17	64	84	130	152	503
Haplotype							
GBHap1		T	T	T	G	T	C
GbHap2		∙	∙	∙	T	C	∙
GbHap3		C	∙	C	∙	∙	∙
GbHap4		∙	C	∙	∙	∙	∙
GbHap5		∙	∙	∙	∙	C	∙
GbHap6		∙	∙	C	∙	∙	T
GbHap7		∙	∙	∙	T	∙	∙

Dots indicate the identity of similar nucleotide bases with the *G. boninense* haplotype 1 (GbHap1) sequence.

**Table 2:** Distribution of seven observed haplotypes, sequence variation and molecular diversity indices among populations of *G. boninense*. Haplotype diversity represent probability that two randomly sampled genes are different. Nucleotide diversity is the average number of nucleotide differences per site in pairwise comparison among gene sequences. As a single population, Tajima’s D was −0.9869 * and Fu’s FS was −2.772 *.

Haplotypes	Populations			
	Miri (*n* = 12)	Asajaya (*n* = 12)	Matu-Daro (*n* = 62)	Balingian (*n* = 31)
GbHap1	0.667	1.000	0.820	0.806
GbHap2	0.167	0	0.098	0.065
GbHap3	0	0	0.049	0.065
GbHap4	0	0	0.016	0
GbHap5	0.083	0	0.016	0
GbHap6	0.083	0	0	0.032
GbHap7	0	0	0.016	0.032
**Sequence Variation**				
Total no. of polymorphic loci (S)	4	0	5	5
Total no. mutation (Eta)	4	0	5	5
Ave. no. nucleotide differences (k)	1.045	0	0.627	0.675
**Molecular Diversity Indices**				
Nucleotide diversity (PiJC)	0.002	0	0.001	0.001
Theta S (per sequence)	1.325	0	1.065	1.252
Number of haplotypes (h)	4	1	6	5
Haplotype diversity (Hd)	0.561	0	0.343	0.351
Tajima’s D	−0.741 *	N/A	−0.955 *	−1.246 *
Fu’s FS	−0.524 *	N/A	−2.272 *	−1.634 *

* Not statistically significant; Tajima’s D *p* > 0.05; Fu’s FS *p* > 0.02. N/A: Not applicable.

**Table 3:** Population subdivision (F_ST_) values and probability test (Chi-square) for population differentiation based on 1000 permutations of the sequence dataset. Genetic subdivision between populations was not detected as indicated by the negative F_ST_ value and the high *p*-value (*p* > 0.05). The low F_ST_ value for Asajaya as with other population was also not statistically significant. *G. boninense* populations within Sarawak showed a homogenous genetic structure and high gene flow (migration index, Nm > 4).

Population	Miri	Asajaya	Matu-Daro	Balingian
Miri	N/A			
Asajaya	0.104 *	N/A		
Matu-Daro	−0.003 *	0.008 *	N/A	
Balingian	−0.014 *	0.048 *	−0.014 *	N/A

* Not statistically significant with *p* > 0.05. N/A: Not applicable.

**Table 4:** Hierarchical analysis of molecular variance (AMOVA) in *G. boninense*. Negative values for variations among populations is regarded as zero. Genetic structure for sampled *G. boninense* population was not detected through AMOVA. Genetic variation was attributed to variation within population.

Source of Variations	Sum of Squares	Variance Components	Percentage of Variations	*p-*Value
Among populations	0.871	−0.001	−0.254	0.503 *
Within populations	34.992	0.310	100.254	

* Not statistically significant with *p* > 0.05.

## Supplementary Materials

Supplementary materials can be found at https://www.mdpi.com/2076-2607/7/10/464/s1. Table S1. Information on *Ganoderma* species used in phylogenetic analysis. Table S2. Pairwise genetic distance among seven haplotypes of *G. boninense* isolated from oil palm plantation in Sarawak with *Tomophagus colossus* (KJ143923) as the outgroup. Table S3. Sampling sites for *G. boninense* basidiomata collection throughout Sarawak.
