# Supplementary material for: Genetic Diversity and Demographic History of Ganoderma boninense in Oil Palm Plantations of Sarawak, Malaysia Inferred from ITS Regions
Source: Microorganisms. 2019 Oct 16;7(10):464. doi: 10.3390/microorganisms7100464 (PMC6843275; doi:10.3390/microorganisms7100464)
Supplement: Supplementary file 1 [file microorganisms-07-00464-s001.pdf]

Table S1. Information on *Ganoderma* species used in phylogenetic analysis.

No.	Species	Voucher/ID	Origin	GenBank® Accession No.	Source	Associated Host or Substrate (If Available)
1.	*G. boninense*	GbHap1	Sarawak, Malaysia	MK713555	This study	*Elaeis guineensis* (oil palm)
2.	*G. boninense*	GbHap2	Sarawak, Malaysia	MK713556	This study	*Elaeis guineensis* (oil palm)
3.	*G. boninense*	GbHap3	Sarawak, Malaysia	MK713557	This study	*Elaeis guineensis* (oil palm)
4.	*G. boninense*	GbHap4	Sarawak, Malaysia	MK713558	This study	*Elaeis guineensis* (oil palm)
5.	*G. boninense*	GbHap5	Sarawak, Malaysia	MK713559	This study	*Elaeis guineensis* (oil palm)
6.	*G. boninense*	GbHap6	Sarawak, Malaysia	MK713560	This study	*Elaeis guineensis* (oil palm)
7.	*G. boninense*	GbHap7	Sarawak, Malaysia	MK713561	This study	*Elaeis guineensis* (oil palm)
8.	*G. boninense*	PER71	Peninsular Malaysia	KM015454	GenBank	*Elaeis guineensis* (oil palm)
9.	*G. boninense*	GB001	Peninsular Malaysia	KX092000	GenBank	*Elaeis guineensis* (oil palm)
10.	*G. boninense*	WD 2028	Japan	KJ143905	GenBank	Unknown
11.	*G. boninense*	WD 2085	Japan	KJ143906	GenBank	Unknown
12.	*G. adspersum*	HMAS86595	England	AY884184	GenBank	Unknown
13.	*G. adspersum*	ITA 39	Finland	EF060011	GenBank	Unknown
14.	*G. adspersum*	Ganoad1	Armenia	JN588579	GenBank	Unknown
15.	*G. adspersum*	Ga-1	Armenia	JN588580	GenBank	Unknown
16.	*G. adspersum*	Ga-2-3	Armenia	JN588582	GenBank	Unknown
17.	*G. adspersum*	Ga-4	Iran	JN588586	GenBank	Unknown
18.	*G. adspersum*	SFC20160115-20	South Korea	KY364254	GenBank	Unknown
19.	*G. adspersum*	HSBU-200894	China	MG279154	GenBank	*Casuarina equisetifolia* (sheoak tree)
20.	*G. adspersum*	ACAM A113	Greece	MG706204	GenBank	*Alnus gltinosa* (common alder tree)
21.	*G. angustisporum*	BJFC Cui 13817	China	NR_158431	GenBank	Unknown
22.	*G. angustisporum*	Cui 14578	China	MG279172	GenBank	*Casuarina equisetifolia* (sheoak tree)
23.	*G. angustisporum*	Cui 16340	China	MG279171	GenBank	*Casuarina equisetifolia* (sheoak tree)
24.	*G. australe*	HMAS86596	England	AY884180	GenBank	Unknown
25.	*G. australe*	C50	Brazil	JQ514105	GenBank	Unknown
26.	*G. australe*	JV 0407/14J	USA	KF605663	GenBank	Unknown
27.	*G. australe*	K-FB2(P).1	Indonesia	KJ654550	GenBank	Unknown

No.	Species	Voucher/ID	Origin	GenBank® Accession No.	Source	Associated Host or Substrate (If Available)
28.	*G. australe*	K-FB2(P).2	Indonesia	KJ654551	GenBank	Unknown
29.	*G. australe*	FLOR 52289	Brazil	KU315203	GenBank	Unknown
30.	*G. australe*	CTRA11	Brazil	KU569540	GenBank	Unknown
31.	*G. australe*	CTRA12	Brazil	KU569541	GenBank	Unknown
32.	*G. australe*	RP57	Brazil	KU569545	GenBank	Unknown
33.	*G. australe*	HUEFS: DHCR411	Australia	MF436675	GenBank	Unknown
34.	*G. australe*	HUEFS: DHCR417	Australia	MF436676	GenBank	Unknown
35.	*G. australe*	JFL 14081134	China	MH106871	GenBank	Unknown
36.	*G. australe*	JFL 14081672	China	MH106872	GenBank	Unknown
37.	*G. australe*	RGM207	Chile	MK322289	GenBank	Blueberry plant
38.	*G. cupreum*	SUT H1	Australia	AY569450	GenBank	Unknown
39.	*G. cupreum*	KR4	India	FJ655467	GenBank	*Prunus armeniaca* (apricot tree)
40.	*G. cupreum*	KR15	India	FJ655469	GenBank	*Terminalia bellerica* (bedda nut tree)
41.	*G. cupreum*	KR6	India	FJ655470	GenBank	*Prunus armeniaca* (apricot tree)
42.	*G. cupreum*	GanoTK7	Cameroon	JN105702	GenBank	Unknown
43.	*G. cupreum*	G133	Malaysia	JN596328	GenBank	Unknown
44.	*G. cupreum*	K24	Malaysia	JN596329	GenBank	Unknown
45.	*G. cupreum*	HMAS130804	China	JX840345	GenBank	Unknown
46.	*G. cupreum*	HMAS99399	China	JX840346	GenBank	Unknown
47.	*G. cupreum*	G84	Unknown	KX055557	GenBank	Unknown
48.	*G. cupreum*	G90	Unknown	KX055560	GenBank	Unknown
49.	*G. curtisii*	238FL	Florida, USA	MG654171	GenBank	Oak, hardwood
50.	*G. destructans*	CMW42157	South Africa	MG020255	GenBank	*Acacia cyclops* (red-eyed wattle)
51.	*G. destructans*	CBS139793	South Africa	NR_132919	GenBank	*Jacaranda mimosifolia* (fern tree)
52.	*G. ellipsoideum*	GACP 14080966	China	NR_160617	GenBank	Decaying wood, conifer
53.	*G. ellipsoideum*	GACP 14080968	China	MH106868	GenBank	Decaying wood, conifer
54.	*G. gibbosum*	AS5.624 type 3	China	AY593856	GenBank	Unknown
55.	*G. gibbosum*	SFC20140702-12	South Korea	KY364260	GenBank	Unknown
56.	*G. lingzhi*	Cui 9166	China	KJ143907	GenBank	Unknown

No.	Species	Voucher/ID	Origin	GenBank® Accession No.	Source	Associated Host or Substrate (If Available)
57.	*G. lingzhi*	SFC20150812-48	South Korea	KY364247	GenBank	Unknown
58.	*G. lingzhi*	Dai12479	China	JQ781864	GenBank	Unknown
59.	*G. lobatum*	JV 1008/32	USA	KF605670	GenBank	Unknown
60.	*G. lobatum*	JV 1008/31	USA	KF605671	GenBank	Unknown
61.	*G. martinicense*	LIP SW-Mart08-55	France	KF963256	GenBank	Unknown
62.	*G. martinicense*	LIP SW-Mart08-44	France	KF963257	GenBank	Unknown
63.	*G. mastoporum*	FRIM 98	Malaysia	AJ627585	GenBank	*Acacia mangium* (brown salwood)
64.	*G. mbrekobenum*	SSP:10	India	KY865253	GenBank	Unknown citrus tree
65.	*G. mbrekobenum*	NGM	India	MH221092	GenBank	Unknown citrus tree
66.	*G. mbrekobenum*	MIN 850481	Ghana	NR_147647	GenBank	Unknown
67.	*G. multipileum*	Dai 9447	China	KJ143914	GenBank	Unknown
68.	*G. multipileum*	CWN 04670	Taiwan	KJ143913	GenBank	Unknown
69.	*G. orbiforme*	Cui 13918	China	MG279186	GenBank	*Casuarina equisetifolia* (sheoak tree)
70.	*G. perzonatum*	SP445985	Brazil	KJ792745	GenBank	Unknown
71.	*G. perzonatum*	SP445987	Brazil	KJ792747	GenBank	Unknown
72.	*G. perzonatum*	SP445990	Brazil	KJ792750	GenBank	Unknown
73.	*G. pfeifferi*	K(M)120818	UK	AY884185	GenBank	Unknown
74.	*G. pfeifferi*	874	Czech Republic	AM906059	GenBank	*Fagus sylvatica* (common beech)
75.	*G. pfeifferi*	GPF2	Poland	JN008874	GenBank	Unknown
76.	*G. philippii*	E7098	Indonesia	AJ536662	GenBank	*Acacia mangium* (brown salwood)
77.	*G. philippii*	E7425	Malaysia	AJ608713	GenBank	*Acacia mangium* (brown salwood)
78.	*G. pseudoferreum*	CATAS-RRI-Gp-01	China	KX454334	GenBank	*Hevea brasiliensis* (rubber tree)
79.	*G. pseudoferreum*	CATAS-RRI-Gp-02	China	KX454335	GenBank	*Hevea brasiliensis* (rubber tree)
80.	*G. ryvardenii*	HKAS58053	Cameroon	HM138671	GenBank	*Elaeis guineensis* (oil palm)
81.	*G. ryvardenii*	HKAS58054	Cameroon	HM138672	GenBank	*Elaeis guineensis* (oil palm)
82.	*G. ryvardenii*	TK31 18S	Cameroon	JN105693	GenBank	*Elaeis guineensis* (oil palm)
83.	*G. ryvardenii*	GanoTK32	Cameroon	JN105698	GenBank	*Elaeis guineensis* (oil palm)
84.	*G. sinense*	XZ-G-C2	Unknown	HQ235634	GenBank	Unknown
85.	*G. sinense*	GDGM25829	China	KC415760	GenBank	Unknown

No.	Species	Voucher/ID	Origin	GenBank® Accession No.	Source	Associated Host or Substrate (If Available)
86.	*G. sinense*	Wei 5327	China	KF494998	GenBank	Unknown
87.	*G. sinense*	zizhi	China	KT906369	GenBank	Unknown
88.	*G. sinense*	Cui 13835	China	MG279193	GenBank	*Casuarina equisetifolia* (sheoak tree)
89.	*G. tornatum*	NPG1	Malaysia	KJ767488	GenBank	Unknown
90.	*G. williamsianum*	Wei 5032	China	KU219994	GenBank	Unknown
91.	*G. williamsianum*	Dai 16809	China	MG279183	GenBank	*Casuarina equisetifolia* (sheoak tree)
92.	*G. zonatum*	FL-02	Florida, USA	KJ143921	GenBank	Palm tree
93.	*G. zonatum*	221FL	Florida, USA	MG654421	GenBank	Unknown
94.	*G. zonatum*	254FL	Florida, USA	MG654426	GenBank	Unknown
95.	*Tomophagus colossus* **(Outgroup)**	TC-02	Vietnam	KJ143923	GenBank	Unknown
96.	*Tomophagus colossus* **(Outgroup)**	UMNFL110	Florida, USA	MG654429	GenBank	Unknown

Table S2. Pairwise genetic distance among seven haplotypes of *G. boninense* isolated from oil palm plantation in Sarawak with *Tomophagus colossus* (KJ143923) as the outgroup. Haplotypes genetic difference of sampled *G. boninense* isolates are low, indicating that they are closely related.

Haplotypes	GbHap 1	GbHap 2	GbHap 3	GbHap 4	GbHap 5	GbHap 6	GbHap 7	Outgroup
GbHap 1	-							
GbHap 2	0.004	-						
GbHap 3	0.004	0.007	-					
GbHap 4	0.002	0.006	0.006	-				
GbHap 5	0.002	0.002	0.006	0.004	-			
GbHap 6	0.004	0.007	0.004	0.006	0.006	-		
GbHap 7	0.002	0.002	0.006	0.004	0.004	0.006	-	
Outgroup	0.117	0.119	0.122	0.120	0.120	0.120	0.117	-

Table S3. Sampling sites for *G. boninense* basidiomata collection throughout Sarawak. For comparison, Kuala Igan samples (n= 6) are included as part of Matu-Daro's population with distance between both location within 40 km. Sampling locations for Miri's population collected from Lambir, Sungai Balim and Sungai Taniku are also situated within 40 to 80 km between plantation location. Sungai Meris and Sungai Liuk representing Balingian's population is located within 5 km from each other.

**No.**	**Population (District/ Sub-District)**	**Location**	**No. of Sample (n)**	**Decimal Degrees (mean)**		**Sampling Date**
				**Latitude**	**Longitude**	
**1**	**Miri**	**Lambir**	**5**	4.135	113.980	7th Jul 2015
		Sungai Balim	5	3.924	113.712	7th Jul 2015
		Sungai Taniku	2	4.424	114.105	7th Jul 2015
2	Asajaya	Sungai Mata	12	1.478	110.663	10th Jul 2015
3	Matu-Daro	Tanjung Alan	56	2.489	111.545	21st Oct 2015
		Kuala Igan	6	2.772	111.726	1st Oct 2018
4	Balingian	Sungai Meris	11	2.964	112.509	29th Mac 2017
		Sungai Liuk	20	2.988	112.548	29th Mac 2017
